# FF-Mamba-YOLO: An SSM-Based Benchmark for Forest Fire Detection in UAV Remote Sensing Images

**DOI:** 10.3390/jimaging12010043

**Published:** 2026-01-13

**Authors:** Binhua Guo, Dinghui Liu, Zhou Shen, Tiebin Wang

**Affiliations:** 1 College of Computer and Control Engineering, Northeast Forestry University, Harbin 150040, China; 2 College of Mechanical and Electrical Engineering, Northeast Forestry University, Harbin 150040, China; 3 College of Aulin, Northeast Forestry University, Harbin 150040, China

**Keywords:** forest fire detection, UAV remote sensing images, Mamba, YOLO, hybrid detection model

## Abstract

Timely and accurate detection of forest fires through unmanned aerial vehicle (UAV) remote sensing target detection technology is of paramount importance. However, multiscale targets and complex environmental interference in UAV remote sensing images pose significant challenges during detection tasks. To address these obstacles, this paper presents FF-Mamba-YOLO, a novel framework based on the principles of Mamba and YOLO (You Only Look Once) that leverages innovative modules and architectures to overcome these limitations. Specifically, we introduce MFEBlock and MFFBlock based on state space models (SSMs) in the backbone and neck parts of the network, respectively, enabling the model to effectively capture global dependencies. Second, we construct CFEBlock, a module that performs feature enhancement before SSM processing, improving local feature processing capabilities. Furthermore, we propose MGBlock, which adopts a dynamic gating mechanism, enhancing the model’s adaptive processing capabilities and robustness. Finally, we enhance the structure of Path Aggregation Feature Pyramid Network (PAFPN) to improve feature fusion quality and introduce DySample to enhance image resolution without significantly increasing computational costs. Experimental results on our self-constructed forest fire image dataset demonstrate that the model achieves 67.4% mAP@50, 36.3% mAP@50:95, and 64.8% precision, outperforming previous state-of-the-art methods. These results highlight the potential of FF-Mamba-YOLO in forest fire monitoring.

## 1. Introduction

Forest fires are considered one of the most severe natural disasters and public crisis events internationally [1]. Annually, millions of hectares of forests are devastated by wildfires, resulting in enormous economic losses and casualties [2]. Furthermore, smoke and greenhouse gas emissions from forest fires not only exacerbate climate change but also damage biodiversity and affect ecosystem functions [3,4]. In arid and semi-arid regions, the threat level of forest fires is particularly severe due to factors such as heat waves, drought, wind, and low humidity [5]. Due to their suddenness and destructiveness [6], forest fires are typically difficult to control. Therefore, rapid and accurate forest fire early warning is crucial for sustainable forest management and ecological protection.

Common early warning systems for forest fires include ground patrols, satellite remote sensing [7,8], and drone surveillance. Ground patrols are limited by factors such as terrain and climate, and require a significant amount of manpower and time, making it difficult to achieve comprehensive coverage. While satellite remote sensing boasts expansive coverage, its real-time capabilities are limited, and cloud interference diminishes its monitoring efficacy in some regions. Additionally, the information processing procedures based on satellite sensor data are relatively complex. In contrast, unmanned aerial vehicles (UAVs) offer strong mobility with minimal terrain and weather constraints. Even when equipped solely with RGB imaging sensors, UAVs are capable of acquiring high-resolution close-range imagery that compensates for the spatial and temporal blind spots of ground and satellite monitoring [9]. These advantages make UAVs highly suitable for rapid forest fire reconnaissance and emergency response. However, unlike general detection tasks, forest fire detection in UAV aerial imagery faces unique challenges. Specifically, forest fire backgrounds are complex and variable, with occlusions and diverse background clutter interference. Second, UAV-captured aerial images exhibit diverse shooting angles and altitudes, leading to significant scale variations in targets in high-resolution images, often presenting multiple targets of different scales simultaneously. This makes accurate detection challenging.

Traditional forest fire detection methods primarily rely on manually extracted fire appearance features. For instance, Bohush et al. [10] proposed a method combining motion estimation, optical flow, and texture analysis based on local binary patterns (LBP) and gray-level co-occurrence matrices (GLCMs) for flame detection in video surveillance systems. Similarly, Rudz et al. [11] developed a precise fire detection model by analyzing flame color and shape. Maeda et al. [12] utilized solar zenith angle and environmental features as characteristics and employed random forest classifiers to identify forest fires, significantly improving fire recognition accuracy. Nazarova et al. [13] achieved precise fire identification by analyzing the spectral characteristics of different regions in satellite imagery. However, such traditional methods often heavily depend on the quality of manually designed features, requiring substantial human effort and time. Additionally, the limitations of manual feature extraction make accurate detection difficult in complex or small fire scenarios, resulting in unstable model detection capabilities and limited generalization across different scenarios.

Deep learning has emerged as a key technology in computer vision in recent years [14,15]. Current deep learning-based object detection models primarily include single-stage, two-stage, and Transformer-based architectures. Two-stage detectors (such as Faster R-CNN [16]) achieve higher accuracy through candidate regions and multi-step optimization, but suffer from high computational overhead and inference latency, making them unsuitable for real-time scenarios such as UAV applications. Single-stage methods (such as the YOLO series [17,18,19,20,21]) employ end-to-end regression processes, achieving an optimal balance between speed and accuracy, but are limited by local receptive fields, constraining their ability to capture long-range dependencies. In contrast, Transformer-based detectors (such as RT-DETR [22]) excel at modeling global and long-range dependencies through self-attention, but their quadratic computational complexity imposes significant computational burdens, limiting their deployment in resource-constrained environments.

State space models (SSMs) [23], represented by Mamba [24], have emerged as promising solutions due to their linear computational complexity for long-range dependency modeling. Initially developed for natural language processing, Mamba’s applications have rapidly expanded to various tasks through efficient modeling of sequence and contextual relationships. Recent research has demonstrated that integrating Mamba into object detection frameworks can leverage its advantages to improve model detection performance [25].

This paper combines Mamba-based modules with YOLOv8 to propose a novel UAV remote sensing forest fire detection framework, providing new insights for addressing these challenges. Our study evaluates FF-Mamba-YOLO against thirteen advanced models on our UFFD, demonstrating its superiority in both accuracy and real-time performance for forest fire detection tasks. The main contributions of this paper are as follows:

(1) Based on the Mamba YOLO architecture, we introduce FF-Mamba-YOLO and construct a UAV remote sensing forest fire dataset (UFFD) containing diverse real-world scenarios, validating the effectiveness and robustness of the proposed model.
(2) We propose CFEBlock, which performs feature enhancement before SSM processing and improves global contextual representation, effectively compensating for SSM’s local modeling capabilities.
(3) We propose MGBlock, which employs a dynamic gating mechanism to adaptively adjust its focus, effectively capturing key features in multiscale environments, thereby improving fire detection accuracy and efficiency.
(4) To improve feature fusion quality, we construct E-PAFPN and introduce the ultra-lightweight dynamic upsampling operator DySample, enhancing object detection accuracy and robustness while maintaining computational efficiency.

## 2. Related Work

### 2.1. Deep Learning for Forest Fire Detection

In recent years, the rapid advancement of deep learning technologies has revolutionized remote sensing forest fire detection. Deep learning-based object detection methods can automatically extract fire features from large amounts of data, offering significant advantages in real-time performance and adaptability. This effectively addresses the real-time requirements, accuracy, and robustness challenges in forest fire detection. With the emergence of convolutional neural networks (CNNs), models such as Faster R-CNN [16], the YOLO series, and Transformer-based models [26,27] have significantly improved detection performance.

Among deep learning-based methods, the YOLO series has remained a mainstream real-time detector in the object detection field. YOLO performs localization and classification simultaneously in a single network through an end-to-end approach, offering good real-time performance and relatively high accuracy, garnering widespread attention in object detection applications. For instance, Lin et al. [28] introduced GhostConv into the YOLOv8 framework for rapid detection of forest fires and smoke. Han et al. [29] employed GhostNetV2 to enhance traditional convolutions in YOLO and integrated an architecture using MHSA attention mechanisms in the backbone, improving accuracy for small-scale forest fire detection while maintaining lightweight characteristics. However, traditional CNNs exhibit significant technical limitations when facing practical application scenarios with substantial scale differences, severe perspective interference, and complex variable backgrounds due to their local receptive fields and hierarchical structural design, lacking adaptability in complex environments.

To address challenges such as local receptive fields and further improve detection performance, the object detection paradigm has recently shifted from CNN-based architectures to Transformer-driven end-to-end frameworks. Yang et al. [30] introduced Swin Transformer into the backbone network, combining it with YOLO’s decoupled head architecture to leverage Transformer’s global modeling capabilities, effectively mitigating feature diversity issues and enabling accurate forest fire identification in complex environments. Li et al. [31] proposed a fire detection framework that combines dual convolution and dual attention mechanisms, introducing image enhancement to improve recognition accuracy with high robustness and generalization capabilities. Despite the success of these methods in improving multiscale flame recognition capabilities, these models face challenges in training convergence, computational costs, and small object detection.

In summary, although deep learning technologies have made progress in UAV remote sensing forest fire detection, challenges remain regarding computational overhead, insufficient adaptability and generalization capabilities, and real-world environmental noise. Therefore, developing an effective detection model is crucial for forest fire detection.

### 2.2. Mamba in Computer Vision

State space model (SSM)-based visual state space models have emerged as a powerful framework in deep learning, particularly in object detection tasks. Liu et al. [32] proposed Vision Mamba, a pure visual backbone based on state space models that employs bidirectional modeling and positional embedding to achieve efficient global visual context representation, demonstrating excellent performance across various visual tasks while significantly reducing computational and memory requirements. Liu et al. [24] introduced VMamba with 2D-selective scan (SS2D), which enhanced its visual processing capabilities. These advances demonstrate the potential of state space-based architectures in computer vision. Initially, research on SSMs in visual tasks primarily focused on image classification and segmentation [33,34]. Recently, however, Mamba-based architectures have been proven to possess tremendous potential in object detection, as demonstrated by Luo et al. [35], who combined novel data augmentation methods with improved Scharr filters and CycleGAN with Mamba-based architectures, achieving state-of-the-art performance in road damage detection. Zheng et al. [36] jointly utilized Transformer and Mamba architectures to address the limitations of existing hyperspectral object detection methods in multiscale representation and global–local feature modeling. Wang et al. [37] proposed novel SSM-based architectures, achieving high-precision cloud detection.

Mamba, with its powerful long-range dependency modeling capabilities and superior linear time complexity characteristics, maintains high accuracy while significantly reducing computational overhead. Therefore, it is regarded as an effective alternative to CNNs and Transformers in resource-constrained scenarios [38]. Inspired by the remarkable achievements of Mamba in the visual domain, integrating Mamba into object detection frameworks can leverage its advantages to achieve efficient detection of remote sensing forest fires.

## 3. Materials and Methods

### 3.1. Datasets

**Figure 1:**
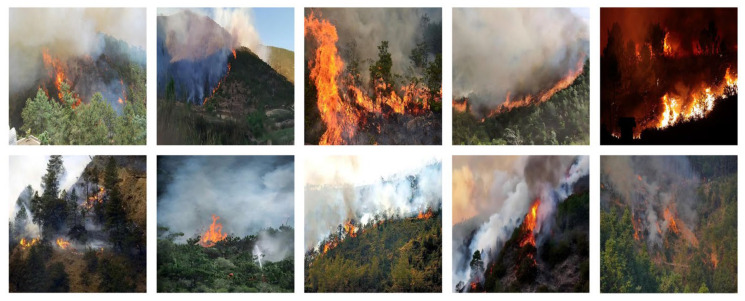
Sample images from the UFFD.

In remote sensing forest fire detection, the dataset quality is pivotal to the precision of forest fire warnings, while datasets play a central role in model development and training. High-quality and diverse datasets can significantly impact the model’s detection accuracy and robustness. However, there remains a dearth of extensive, high-caliber real-world forest fire remote sensing data. To address the limitations of existing forest fire remote sensing datasets, in this paper, we construct a UAV Remote Sensing Images dataset for Forest Fire Detection based on real scenarios that is named UFFD, as illustrated in Figure 1. We manually collected and carefully screened clear standard images from the internet, ultimately obtaining 6548 real-world UAV forest fire images of varying scales, shapes, and sizes. These images encompass diverse scenarios, including a range of terrain types (mountains, plains, and hills), different lighting intensity levels (spanning daytime, dusk, and nighttime), and varying numbers of forest fire occurrences, providing a comprehensive view of various fire situations, thereby enhancing the model’s generalization capability.

**Figure 2:**
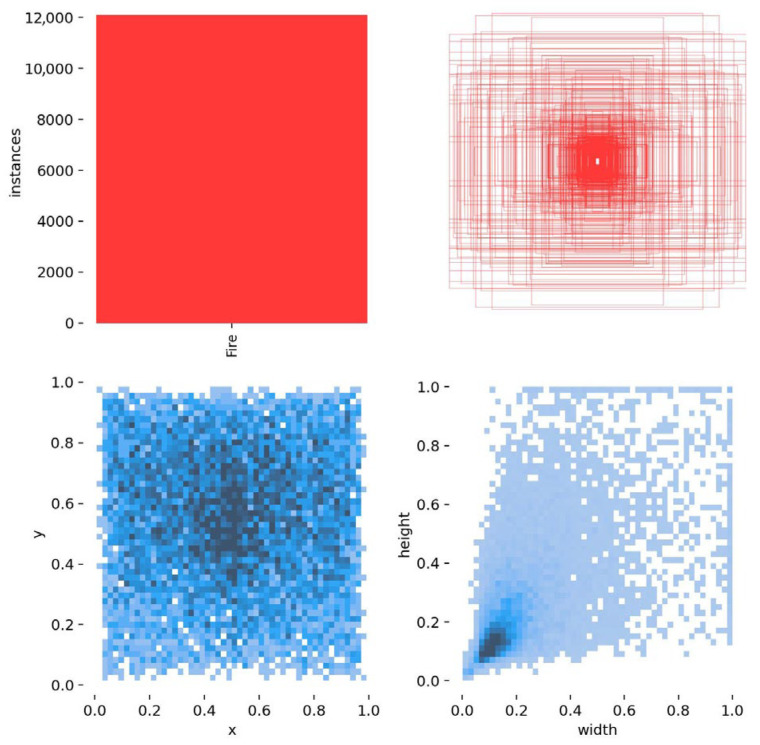
Visual distribution map of the UFFD.

Figure 2 shows that, regarding bounding box statistics, the dataset contains approximately 12,000 bounding boxes with extensive distribution. This indicates that our dataset is richly annotated and reflects the multiscale characteristics of the targets, providing ample ground truth data for the model to identify fire features. All images are resized to a uniform size of 640 × 640 pixels. In this study, the dataset is divided into training, validation, and test sets with a distribution ratio of 8:1:1, resulting in 5238, 655, and 655 images, respectively. This partitioning strategy ensures sufficient data availability for model training.

### 3.2. Overall Architecture

**Figure 3:**
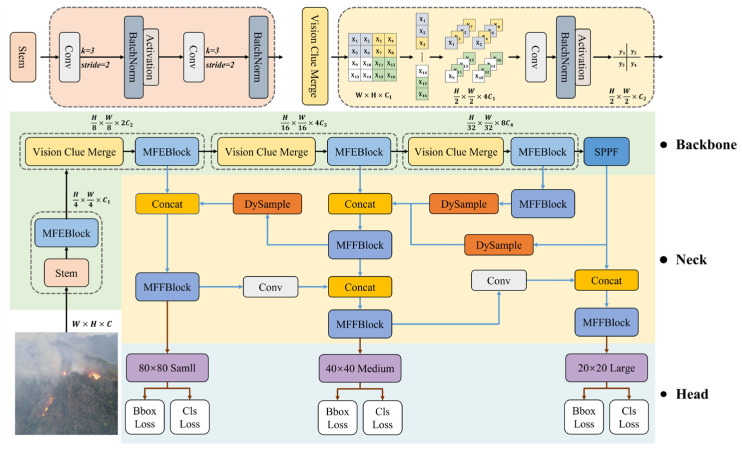
Overall architecture of FF-Mamba-YOLO.

This paper proposes FF-Mamba-YOLO, a Mamba-based YOLO framework that integrates the advantages of Mamba and CNNs to meet the requirements for efficient forest fire monitoring. The model consists of three main components: the backbone network, neck, and head. Figure 3 illustrates the overall architecture of the proposed FF-Mamba-YOLO model.

The backbone network adopts a state-space model-based architecture. The method involves downsampling the input image I ∈ *W × H × C* through the stem to obtain a 2D feature map with resolution $\frac{H}{4}$ × $\frac{H}{4}$, followed by a series of MFEBlock and Vision Clue Merge [39] combination structures for progressive feature learning. Finally, pooling operations are performed through the Spatial Pyramid Pooling Fusion (SPPF) module to obtain multiscale target features. This design provides rich feature information for subsequent object detection tasks while maintaining efficient computational performance.

In the neck network section, an extended PAFPN design is adopted, which enhances the PAFPN structure by introducing a new feature map P5′ at the P5 layer to capture more gradient-rich information flow. To further improve the upsampling quality, we introduce the DySample dynamic upsampling module, which achieves adaptive feature interpolation through learnable offsets, providing more precise spatial information upsampling while significantly reducing computational complexity.

Finally, the aggregated multiscale feature maps are passed to a decoupled detection head, which accomplishes precise object detection through two parallel branches of bounding box regression and classification prediction, thereby achieving target position estimation and category determination.

### 3.3. Visual State Space Model

#### 3.3.1. State Space Model

In recent years, state space models have attracted substantial research attention. Mamba is a state space model (SSM) that has emerged as a compelling alternative by enhancing sequence modeling capabilities while addressing the computational efficiency issues of Transformers in modeling long sequences of state spaces.

SSMs are typically regarded as linear time-invariant (LTI) systems. Given a continuous input $x(t)\in R^{L}$, the SSM’s hidden state $h(t)\in R^{N}$ transforms it into an output $y(t)\in R^{L}$, which can be represented by the following linear ordinary differential equations (ODEs):

(1) $$h^{\prime }(t)=Ah(t)+Bx(t)$$

(2) y(t)=Ch(t)

where $A\;{\in R}^{N\times N}$ represents the state transition matrix, and $B,C\;{\in R}^{N}$ denote the input matrix and output matrix, respectively.

In deep learning applications, input data is typically discrete; for example, it may take the form of image data. Therefore, continuous-time SSMs must be discretized in advance. By applying the zero-order hold principle to the input signal, an equivalent discrete-time representation can be achieved, which can be expressed as follows:

(3) $$\bar{A}=e^{∆A}$$

(4) $$\bar{B}={(e}^{∆A}-I)A^{-1}B$$

where $\bar{A}$ and $\bar{B}$ correspond to the discrete forms of **A** and **B**, respectively; ∆ represents the discrete time step; and **I** is an identity matrix.

After discretization, the input $x(k)\in R^{L}$ is transformed into output $y(k)\in R^{L}$ through the hidden state h(k). The discretized state space model can be described as follows:

(5) $$h_{k}=\bar{A}h_{k-1}+\bar{B}x_{k}$$

(6) $$y_{k}=Ch_{k}$$

This process facilitates the integration of Mamba into deep learning frameworks for computational purposes.

#### 3.3.2. Two-Dimensional Selective Scan for Vision Data (SS2D)

**Figure 4:**
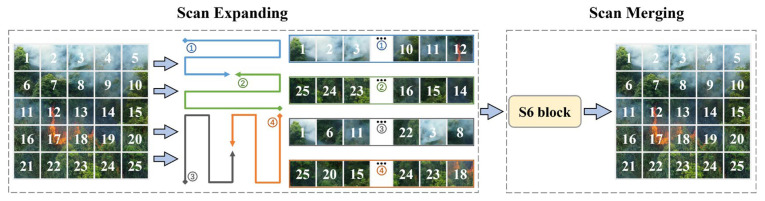
The illustration of SS2D operation.

The S6 module, as the core of Mamba, achieves global receptive field, dynamic weights, and linear complexity, enabling Mamba to perform well in natural language processing (NLP) tasks. However, it performs causal processing on input data, meaning it can only capture information from the scanned portion, which makes it difficult to capture potential connections between unscanned regions when processing visual data. To address this challenge, VMamba [24] introduced an SSM-based module that serves as a visual center, facilitating efficient learning of visual representations, and proposed the SS2D mechanism. An illustration of SS2D operation is presented in Figure 4. SS2D comprises three main stages: cross-scanning, selective scanning based on S6 modules, and cross-fusion. Specifically, SS2D segments the input image into multiple blocks and unfolds these blocks into sequences along four different scanning paths (i.e., cross-scanning). Subsequently, it processes each block sequence in parallel through different S6 modules and reorganizes and fuses the outputs of individual sequences to generate the final output feature map. The operation of SS2D can be represented as follows:

(7) $$x_{i}=expand(x,i)$$

(8) $${\bar{x}}_{i}=S6(x_{i})$$

(9) $$\bar{x}=merge({\bar{x}}_{1},{\bar{x}}_{2},{\bar{x}}_{3},{\bar{x}}_{4})$$

where $i\in \left\{1,2,3,4\right\}$ represents four different scanning directions, while expand( · ) and merge( · ) denote the scanning expansion and merging operations, respectively. Through this design, SS2D efficiently captures both local and global context of images through multi-directional scanning, enabling each pixel in the feature map to integrate information from different positions while maintaining linear complexity and covering the global receptive field, offering an enhanced performance in visual tasks. For further details regarding the proof and derivation, please refer to the original paper by VMamba [24].

### 3.4. The Proposed Model

#### 3.4.1. Mamba-Based Modules

**Figure 5:**
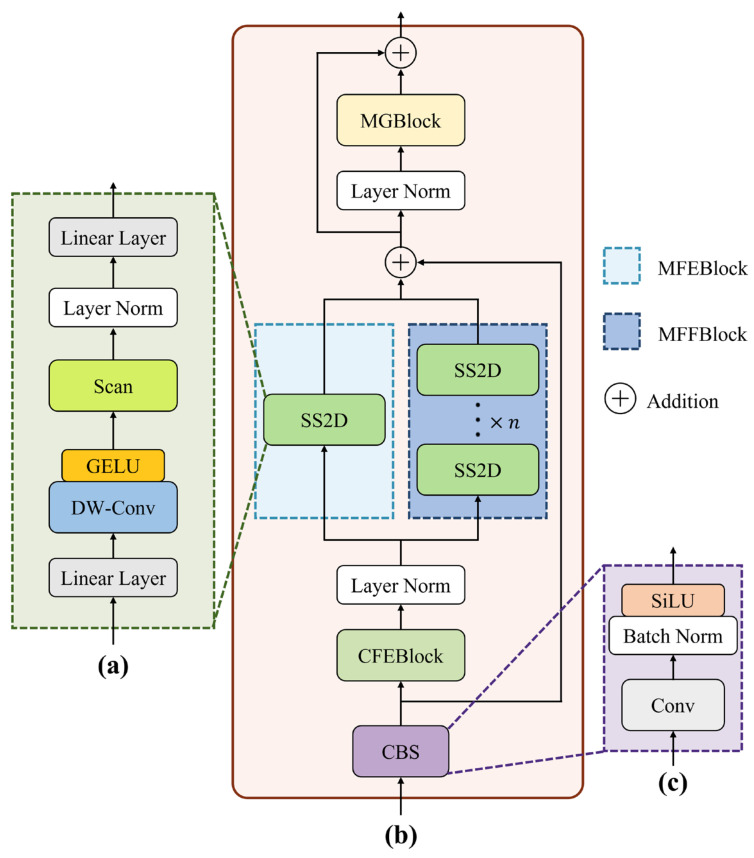
(**a**) Detailed structure diagram of SS2D. (**b**) Architecture diagram of MFEBlock and MFFBlock. (**c**) Detailed structure diagram of CBS.

The complex background of UAV remote sensing forest fires poses significant challenges for object detection tasks. While traditional convolutional neural networks (CNNs) are effective for many computer vision tasks, determining long-range relationships between targets and capturing their complex features remain formidable challenges due to their inherent spatial invariance and limited receptive fields. In contrast, SSMs can effectively establish long-range dependencies in images through state space modeling, enabling effective capture of key features even under complex conditions such as dense target arrangements, occlusions, and background interference. Therefore, integrating Mamba modules based on state space models (SSMs) into the YOLOv8 network significantly enhances the detection capability for UAV remote sensing forest fire images. Inspired by Wang et al. [39] and Liu et al. [24], this paper employs the SS2D module as the core component of the state space model (SSM) and proposes Mamba-based Feature Extraction Block (MFEBlock) and Mamba-based Feature Fusion Block (MFFBlock) modules based on the state space model (SSM) mechanism (shown in Figure 5).

MFEBlock serves as the core component for extracting multiscale features in FF-Mamba-YOLO. Leveraging optimized state space models (SSMs) enables the backbone network to effectively utilize state space modeling, significantly enhancing the efficiency and comprehensiveness of multidimensional image feature capture. Furthermore, MFEBlock adopts a configurable branch structure that provides flexible parameter configuration for feature extraction tasks at different levels while maintaining computational efficiency. This design not only fully exploits state space modeling but also optimizes computational resource allocation, maintaining the light weight of the model and ensuring that it has efficient feature extraction capabilities.

In the neck network, MFFBlock is responsible for fusing multiscale feature information from different levels. Building upon MFEBlock, MFFBlock employs multiple SS2D modules in series with fixed branch usage, providing stronger long-range dependency capture capabilities. It can effectively integrate multiscale and diverse features from the backbone network, achieving refined fusion of multiscale features. This design significantly improves the model’s ability to process information in complex environments, providing richer feature representations for subsequent object detection tasks.

These improvements effectively determine long-range correlations between flames at different positions and scales while capturing distinctive features, maintaining linear computational complexity and significantly enhancing the model’s robustness and stability.

#### 3.4.2. Contextual Feature Enhancement Block

**Figure 6:**
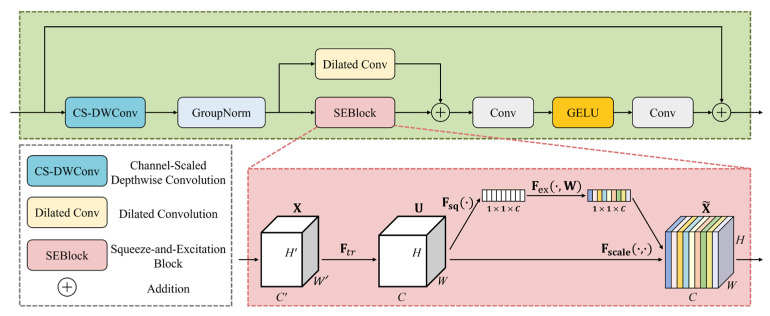
The structure of the CFEBlock module.

In forest fire remote sensing images, targets exhibit significant scale variations and complex spatial distribution characteristics. While Mamba architectures based on state space model (SSM) mechanisms can effectively capture long-range spatial dependencies, challenges remain with regard to local feature extraction and feature enhancement. To address this challenge, this paper proposes the Contextual Feature Enhancement Block (CFEBlock) module. The schematic diagram of this module is shown in Figure 6.

The module begins by performing Channel-Scaled Depthwise Separable Convolution (CS-DSConv). Compared with traditional depthwise separable convolution [40], it implements a learnable channel-level weight scaling mechanism through broadcast multiplication, enabling the network to adaptively adjust the importance of different channels through global training, achieving intelligent feature selection. This design effectively preserves the detailed information of small targets without altering the spatial resolution. Furthermore, this mechanism enables the model to effectively reduce computational costs and parameter count while improving the model’s generalization capability. Subsequently, GroupNorm normalization [41] is applied to replace traditional BatchNorm, improving training stability and memory usage efficiency.

To more effectively capture key and rich feature information in images and expand the receptive field, CFEBlock adopts a dual-branch parallel processing architecture. Specifically, the left branch is equipped with a dilated convolution [42], which allows the module to effectively expand the receptive field to encompass a larger context without increasing parameter count or computational overhead. The right branch introduces a Squeeze-and-Excitation block (SEBlock) [43], enabling the network to perform dynamic channel feature recalibration. Through this approach, the model can adaptively reweight channel features, emphasizing informative channels and suppressing less useful ones, further enhancing the model’s global context representation capability. The outputs of both branches are merged by concatenating their feature maps along the channel dimension.

Subsequently, the feature maps are enhanced through activation functions to improve feature expressiveness. Notably, the activation function employs non-linear Gaussian error linear unit (GELU), better preserving and distributing information flow and enabling the model to learn more complex feature representations. Finally, the module fuses the processed features with the original input features through residual connections, ensuring gradient flow and information preservation, thereby avoiding network degradation issues and enabling the model to learn effective features from images.

CFEBlock performs multiscale feature extraction and feature enhancement on input features before SSM processing, providing rich multiscale information representation for SS2D, enhancing the model’s global context representation capability, and significantly improving the model’s ability to process complex features, demonstrating superior performance in challenging environments.

#### 3.4.3. Multiscale Gated Block

Forest fire remote sensing images exhibit complex target characteristics and face challenges such as occlusions and background interference. On the other hand, flame positions and structures are highly dynamic, with significant differences between fire targets of different scales, making it difficult for traditional feature processing mechanisms to effectively adapt to these complex multiscale conditions. To address these challenges, this paper proposes the Multiscale Gated Block (MGBlock) module. This module adopts a dynamic gating mechanism and multibranch structure design, effectively solving the adaptability limitations of traditional methods in complex fire scenarios and providing a more robust and efficient multiscale feature processing solution for forest fire detection.

**Figure 7:**
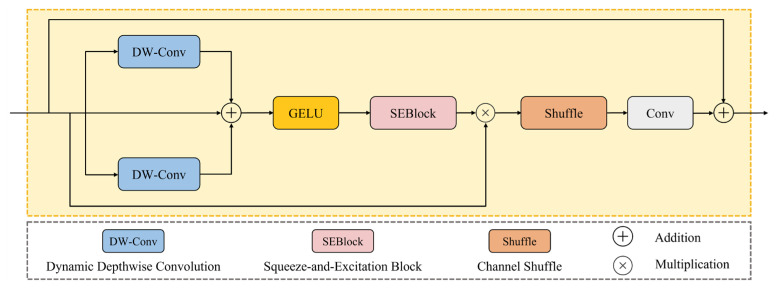
The structure of the MGBlock module.

Specifically, inspired by the application of Gated MLP [44] in natural language processing, we introduce a dynamic gating mechanism in MGBlock. This mechanism can adaptively regulate information flow paths and addresses key challenges faced by original MLPs, such as excessive parameter count and lack of spatial structural information. As shown in Figure 7, the MGBlock module divides input features into two branches for feature processing and controlling information flow intensity, respectively.

While standard convolutions are effective for many computer vision tasks, they lack adaptive processing capabilities for fire targets of different scales due to their inherent spatial invariance and limited receptive fields, presenting limitations in remote sensing forest fire detection. To address this, the feature processing branch is equipped with two parallel depthwise convolution modules that more effectively capture multiscale feature information in images through parallel processing. Specifically, 3 × 3 depthwise convolution focuses on capturing local detail features (such as flame edges and texture details), while 5 × 5 depthwise convolution captures broader spatial contextual information (such as flame shapes and structures). The outputs of the two convolution branches are then fused through an adaptive weight fusion mechanism. This design enables the network to better adapt to different input scenarios and task requirements, dynamically adjusting the importance of features at different scales according to task demands, thereby improving detection accuracy and model flexibility.

The fused features are then merged with the original input features through residual connections, allowing the model to understand and integrate features of different dimensions in the image, achieving effective integration of multiscale features. The feature representation capability is further enhanced through GeLU activation functions.

To further improve the network’s representational capability, MGBlock processes the fused features through a lightweight SE channel attention mechanism. The processed features are merged with the gating branch through element-wise multiplication, enabling the module to more effectively capture key feature information.

Subsequently, the ChannelShuffle [45] operation is performed, enhancing information interaction between different channel groups and significantly improving the model’s representational capability with minimal computational overhead. The features are then refined through 1 × 1 convolution with global features to blend channel information.

Finally, the module’s output is merged with the original input features through adaptive residual connections. When input and output dimensions differ, 1 × 1 convolution is used for dimension adjustment; otherwise, identity mapping is employed directly, ensuring gradient flow stability and feature information integrity.

### 3.5. Multiscale Feature Fusion

#### 3.5.1. E-PAFPN

PAFPN [46] improves performance through path augmentation and aggregation, systematically refining from semantically rich features to basic features and implementing the reverse process. This method enhances detection accuracy on remote sensing image datasets while maintaining real-time performance. However, the traditional PAFPN’s P5 branch only draws one path from the SPPF output. Although SPPF can rapidly extract multiscale features, pooling operations often smooth high-frequency details, losing critical information during feature fusion, which may affect detection accuracy.

**Figure 8:**
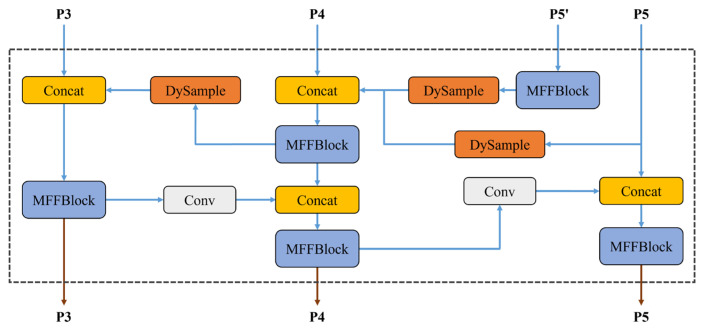
Structure of E-PAFPN.

To overcome these limitations, we propose E-PAFPN, which enhances the structure by introducing a new feature map P5′ at the P5 layer. This feature map undergoes MFFBlock processing and DySample upsampling before participating in P4 layer feature fusion, better preserving the long-range dependencies of state space modeling and fine-grained texture structures. The structure of E-PAFPN is shown in Figure 8.

Such improvements can better utilize fine-grained textures, edges, and long-range dependency information that have not undergone SPPF multiscale pooling. Although this introduces a modest computational overhead, it enables the capture of richer information flow gradients and long-distance relationships between different levels. This information provides broader contextual understanding for the P4 layer, enhancing the representational capability of the feature pyramid network, which is crucial for improving feature fusion quality and detection accuracy in flame detection.

#### 3.5.2. DySample

**Figure 9:**
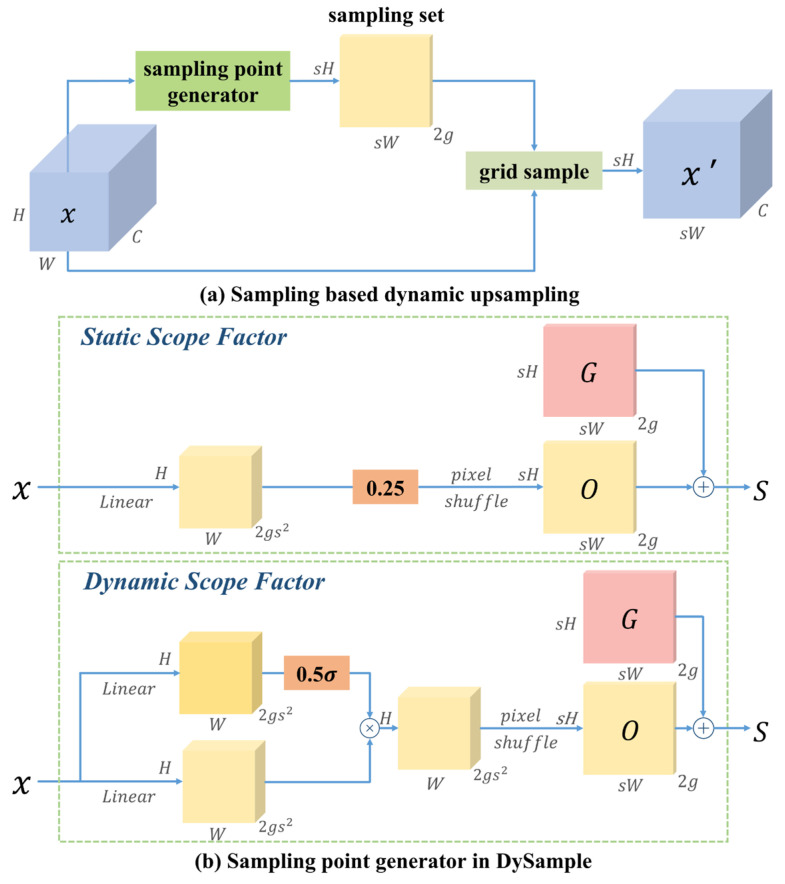
Structure of the DySample model.

The upsampling layer in YOLOv8 typically employs nearest neighbor interpolation (Nearest). The problem with this approach lies in its disregard for smooth transitions between pixels, in which only a small number of surrounding pixels are used for prediction, which may lead to the loss of important image details. Furthermore, the upsample method requires substantial computational resources, potentially limiting the model’s ability to maintain lightweight performance in UAV forest fire detection. To overcome these challenges, this study introduces DySample [47] during the feature fusion stage. This module improves resource efficiency by bypassing dynamic convolution and adopting point sampling methods for upsampling. Simultaneously, DySample’s learnable offset mechanism enables more precise handling of complex spatial relationships in forest scenarios, reducing target misalignment and feature loss caused by improper upsampling. This design enables the model to better adapt to complex and variable scenarios in UAV forest fire detection, enhancing detection accuracy and robustness for multiscale targets while maintaining computational efficiency. Therefore, the DySample module is particularly suitable for object detection in scenarios with diverse intricacies, as described in this paper. Figure 9 illustrates the sample-based dynamic upsampling and module design associated with DySample.

Given a feature map *X* of size $C\times H_{1}\times W_{1}$ and a sampling set ***S*** of size $2\times H_{2}\times W_{2}$, where the first dimension of 2 represents the x and y coordinates, the grid_sample function resamples *X* using the coordinates from sampling set *S*. This is achieved by applying bilinear interpolation methods, generating a new feature map *X*′ of size *C* × *H*_2_ × *W*_2_. This process is defined as follows:

(10) $$X^{\prime }=grid\_sample(X,\;S)$$

Let the upsampling scale factor be *s*, while the feature map *X* has the dimensions *C* × *H* × *W*. A linear layer with input and output channel numbers of *C* and $2s^{2}$, respectively, is used to generate an output offset *O* of size $2s^{2}\times \;H\;\times \;W$. This is then reshaped to 2 × *sH* × *sW* through pixel shuffling. Subsequently, the sampling set *S* is obtained from the superposition of offset *O* with the original sampling grid *G*. This process is defined as follows:

(11) O=linear(X)

(12) S=G+O

where the reshaping operation is omitted. Finally, an upsampled feature map *X*′ of size *C* × *sH* × *sW* can be generated.

This method dynamically adjusts each point during upsampling by learning offsets to more accurately restore the foreground features of flame targets, thereby improving the model’s spatial perception capability for more accurate localization and identification of real fire locations.

### 3.6. Metrics for Evaluating Object Detection

To comprehensively evaluate the performance of the proposed method, this study employs a series of evaluation metrics, including model parameters (Params), giga floating-point operations (GFLOPs), precision (P), recall (R), and mean average precision (mAP). Specifically, the parameters metric quantifies the model’s storage requirements, providing insight into its memory footprint. The GFLOPs metric measures the volume of computational operations, reflecting the model’s processing intensity. Precision (P) measures the accuracy of positive predictions, representing the proportion of true positive samples among samples predicted as positive by the model. Recall (R) measures the model’s ability to identify all relevant instances in the dataset, representing the proportion of correctly predicted positive samples among all positive samples. mAP is a standard metric for evaluating object detection model performance, and is used to measure the average accuracy of predicted target localization and classification. It is calculated by averaging the AP values across all classes, where AP summarizes the precision–recall curve for each class at different IoU thresholds. Specifically, mAP@50 represents the mAP value at an IoU threshold of 0.5, while mAP@50:95 represents the average mAP value at IoU thresholds from 0.5 to 0.95 (with a step size of 0.05). The calculation formulas for these metrics are as follows:

(13) $$P=\frac{TP}{TP+FP}$$

(14) $$R=\frac{TP}{TP+FN}$$

(15) $$AP=\int_{0}^{1}P(R)dR$$

(16) $$mAP=\frac{1}{N}\sum_{i=1}^{N}{AP}_{i}$$

In the proposed formulas, TP represents the number of true positive samples correctly predicted as positive by the model, FP represents the number of false positive samples incorrectly predicted as positive by the model, FN represents the number of false negative samples incorrectly predicted as negative by the model, and N represents the number of classes.

## 4. Results

### 4.1. Experimental Environment and Parameters

The experiments were conducted using PyTorch 2.0.0 as the deep learning framework, with Python 3.8 as the programming environment. All experiments were performed on RTX 4090 GPU (24 GB VRAM) paired with Intel(R) Xeon(R) Platinum 8481C CPU, running the Ubuntu 20.04 operating system.

The detailed training hyperparameter configuration used in this experiment is presented in Table 1. During training, we employed the Stochastic Gradient Descent (SGD) optimizer with an initial learning rate set to 0.01. To stabilize training, a weight decay of 0.0005 and a momentum factor of 0.937 were applied. The model was trained for 150 epochs with a batch size of 4. Input images were uniformly resized to 1024 × 1024-pixel resolution. The data loading process utilized eight worker threads to enhance preprocessing efficiency.

### 4.2. Comparison Between YOLOv8s and FF-Mamba-YOLO

**Figure 10:**
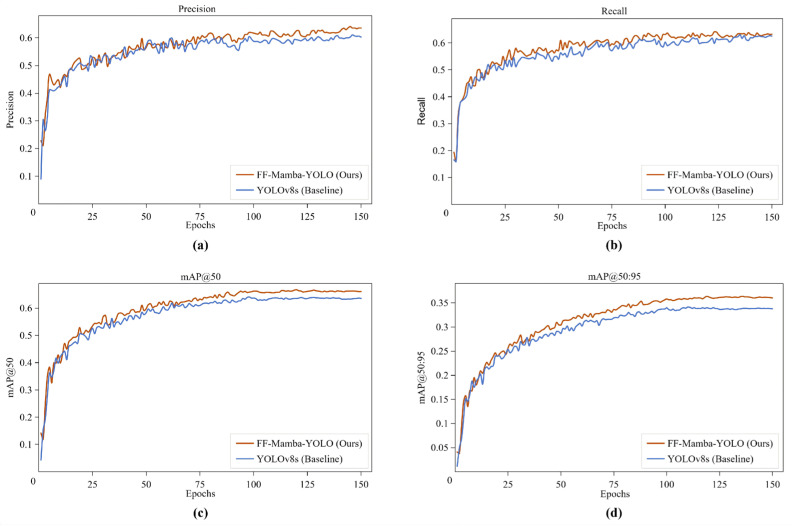
Training curve of FF-Mamba-YOLO and YOLOv8s. (**a**) Precision. (**b**) Recall. (**c**) mAP@50. (**d**) mAP@50:95.

To evaluate the performance of the proposed model and intuitively demonstrate the performance evolution during training, we trained and validated FF-Mamba-YOLO and the baseline model YOLOv8s on the UFFD. The comparative results on the validation set are shown in Figure 10. During the initial training stage, the four core evaluation metrics of both models rapidly improved and converged after approximately 100–150 epochs. Ultimately, FF-Mamba-YOLO outperformed the baseline YOLOv8s in precision, recall, mAP@50, and mAP@50:95. These results demonstrate the superior performance of FF-Mamba-YOLO in detecting forest fires from UAV perspectives.

### 4.3. Comparison Experiment

To comprehensively validate the superior performance of FF-Mamba-YOLO and the effectiveness of its improvements, we conducted an extensive comparative analysis of the proposed UFFD, comparing it with thirteen advanced object detection algorithms. The comparison encompasses various detection paradigms, including two-stage detectors (e.g., Faster R-CNN [16]), mainstream models from the YOLO series, Transformer-based RT-DETR [22], and Mamba-based Mamba-YOLO [39]. All experiments were conducted under identical experimental conditions to ensure fair comparison. The experimental results on the test set are presented in Table 2.

The experimental results demonstrate that FF-Mamba-YOLO achieves 67.4% mAP@50, 36.3% mAP@50:95, and 64.8% Precision, achieving the best performance among all models and showcasing its exceptional accuracy in UAV remote sensing fire detection. Although FF-Mamba-YOLO’s Recall of 62.1% is slightly lower than the best result, its comprehensive precision, efficiency, and stability make it highly suitable for UAV-based real-time forest fire monitoring.

Faster R-CNN, as a two-stage detector, demonstrates 62.0% mAP@50. However, due to its requirement for region proposal generation followed by object classification and bounding box regression, it incurs substantial computational overhead, with its parameter count and computational load reaching 28.3M and 157.0 GFLOPs, respectively, making it unsuitable for real-time applications and challenging to adapt to object detection tasks in UAV remote sensing images.

In contrast, models in the YOLO series, as single-stage detectors, exhibit superior real-time performance while maintaining relatively high detection accuracy, making them more suitable for UAV detection tasks. For instance, YOLOv9s achieves 65.1% mAP@50 and 62.2% precision, with a lower parameter count and GFLOPs compared with two-stage methods. However, they are relatively limited in advanced feature fusion and contextual modeling, making them susceptible to complex backgrounds and significant scale variations, which restricts their application in complex object detection tasks.

Lightweight models, such as YOLOv8n, utilize only 3.0M parameters and 8.1 GFLOPs, demonstrating excellent model efficiency. However, the reduced parameter count and simplified architecture further limit their representational capability and contextual modeling depth, resulting in lower detection accuracy with mAP@50 of only 62.7% and mAP@50:95 of only 32.9%. Their capability to capture complex details such as flames from UAV perspectives remains limited.

Transformer-based models, such as RT-DETR, achieve 59.6% mAP@50 and 30.3% mAP@50:95 due to their effective capture of long-range dependencies in images through global attention mechanisms and improved detection accuracy for large-scale targets. However, their detection capability for small targets is limited, while their parameter count and GFLOPs are significantly higher than those of the proposed model. Therefore, their practicality in resource-constrained, complex background UAV forest fire detection tasks is constrained.

Mamba-based models achieve the fourth highest mAP@50 and mAP@50:95, at 65.0% and 35.1%, respectively, showing advantages over YOLO series models and Transformer-based models. However, they are limited by their simplified architecture, and thus, their multiscale representation and local detail modeling remain insufficient, with limited robustness when facing targets of different sizes and increased susceptibility to false and missed detections in complex backgrounds. Therefore, their practicality in remote sensing forest fire detection tasks remains limited.

In summary, our results demonstrate that FF-Mamba-YOLO exhibits significant advantages in remote sensing forest fire detection tasks. The proposed structural and algorithmic optimizations achieve a favorable balance between performance and computational resources, reflecting the model’s practicality and reliability in real forest fire scenarios and providing a novel technical solution for real-time object detection.

### 4.4. Ablation Experiments

#### 4.4.1. Ablation Study on DySample

The DySample module introduced in this study significantly enhances the representational capability of upsampled feature maps for remote sensing forest fire features through adaptive sampling mechanisms. To demonstrate the advantages of DySample in FF-Mamba-YOLO, we conducted systematic experiments to evaluate the impact of different upsamplers. As shown in Table 3, the experiments demonstrate the influence of upsamplers on detection performance and computational efficiency.

The experimental results demonstrate that DySample’s performance surpasses all of the compared upsamplers. After integrating DySample, the model achieves significant performance improvements under conditions in which the computational cost remains nearly unchanged (20.1 GFLOPs) and the parameter count remains essentially identical (9.4M). Specifically, mAP@50 increases to 67.4%, representing an improvement of approximately 1% over nearest neighbor and bilinear interpolations; mAP@50:95 reaches 36.3%; and precision increases to 64.8%. Although the recall is 62.1%, the significant improvements in Precision and both mAP metrics highlight the effectiveness of DySample in enhancing detection capabilities.

Nearest neighbor and bilinear interpolations achieve recall values of 64.6% and 62.6%, respectively, showing moderate improvements compared with the DySample method. However, they perform poorly regarding detection accuracy, with lower mAP@50 values of 66.5% and 66.2%, mAP@50:95 values of 35.6% and 35.2%, and precision values of 59.1% and 62.4%, all inferior to the DySample method.

In contrast, another dynamic upsampler CARAFE [48] achieves both high precision and recall of 62.7% and 62.4%, respectively, but mAP@50 is only 66.3% and mAP@50:95 is 35.8%, showing no substantial improvement compared to the nearest and bilinear methods. Furthermore, CARAFE increases computational load and model size to 20.7 GFLOPs and 9.8M parameters, respectively, resulting in higher resource requirements and inferior cost-effectiveness compared with DySample.

Overall, DySample upsampling provides the optimal balance between performance and efficiency. It delivers significant improvements in Precision and mAP without causing computational or parameter overhead. Therefore, DySample is selected as the upsampler for the final model configuration. This choice is significant for recovering detailed information of forest fires in UAV aerial images, facilitating better recognition of small targets and complex texture features by the model.

#### 4.4.2. Component-Wise Ablation Analysis

To systematically evaluate the contribution of each component to model performance, we conducted a series of ablation experiments on the UFFD.

- Individual module performance.

Table 4 presents the results of individual module ablation experiments. After introducing CFEBlock, the model’s recall increased by 3.7%, mAP@50 increased by 1.7%, and mAP@50:95 increased by 1.9%, demonstrating the effectiveness of this module. This improvement benefits from CFEBlock’s effective enhancement of the ability to process complex details of small targets through CS-DSConv. Simultaneously, dilated convolution expands the receptive field, learning richer feature information and improving the model’s adaptability in complex scenarios.

MGBlock introduces a dynamic gating mechanism, enabling better adaptation to the detection requirements of multiscale targets and complex shape variations in UAV images. This results in improvements across all model metrics, with mAP@50 increasing by 1.2%; mAP@50:95 increasing by 1.4%; and precision and recall increasing by 0.1% and 1.4%, respectively, demonstrating its effectiveness in extracting complex features.

The addition of E-PAFPN improves mAP@50 by 0.9% and mAP@50:95 by 1.1%. This indicates that E-PAFPN captures richer features by introducing new feature maps, effectively improving feature fusion quality.

DySample significantly enhances the representation capability of upsampled feature maps for flame features through adaptive sampling mechanisms, improving mAP@50 and mAP@50:95 by 0.4% and 0.9%, respectively. Notably, recall demonstrates more significant improvement, increasing by 4.6%, alleviating the problem of missed detections during the detection process.

- Inter-module synergy effects.

As shown in Table 5, joint improvements between modules can further optimize the model’s detection performance. The combination of E-PAFPN and DySample can capture richer information flow gradients and long-range relationships between different levels while maintaining controllable computational load, significantly improving the model’s feature fusion quality, thereby increasing mAP@50 to 65.7%, mAP@50:95 to 35.1%, and precision to 62.1%.

Although the integration of CFEBlock and MGBlock is accompanied by increases in parameter count and GFLOPs, it significantly enhances the model’s detection performance, improving mAP@50 to 66.2% and mAP@50:95 to 35.4%. These performance metrics are key factors determining the applicability scope and capabilities of algorithms in practical applications, demonstrating the practical value of the proposed modules.

After integrating E-PAFPN and DySample, respectively, the combination of CFEBlock and MGBlock continues to surpass previous variants in mAP, demonstrating improved multiscale context capture and upsampling fidelity.

The final FF-Mamba-YOLO model achieves optimal results across all evaluation metrics, with mAP@50 achieving a significant improvement of 67.4%, while mAP@50:95 and precision reach 36.3% and 64.8%, respectively. The ablation study results confirm that each proposed component generates synergistic benefits, ultimately forming a balanced solution that fully validates the effectiveness of the designed algorithms and structures.

### 4.5. Generalization Experiment

To further validate the superiority and robustness of the proposed FF-Mamba-YOLO model in UAV remote sensing forest fire image object detection, we conducted a generalization experiment. For this purpose, we extensively validated the proposed model against three top-performing architectures (YOLOv9s, YOLOv13s, and Mamba-YOLO) on the FlameVision [49] dataset. The FlameVision dataset is a comprehensive high-altitude image dataset specifically designed for detecting and classifying wildfires. We adopted the object detection portion of the FlameVision dataset and annotated 4500 images, which were randomly divided into training, validation, and test sets in an 8:1:1 ratio. The epoch was set to 100, with other hyperparameters consistent with the training configuration of comparative experiments.

As shown in the experimental results in Table 6, FF-Mamba-YOLO achieves mAP@50, mAP@50:95, and precision of 95.5%, 72.4%, and 90.8%, respectively, all significantly outperforming other advanced algorithms. This demonstrates that our model exhibits excellent generalization capabilities across different data distributions, as well as superior feature extraction capabilities and multiscale target adaptability, enabling more accurate flame identification in complex and variable remote sensing scenarios. This further confirms the practicality and reliability of the proposed model in real-world applications.

### 4.6. Visualization and Comparative Analysis

#### 4.6.1. Experimental Results Visualization Analysis

**Figure 11:**
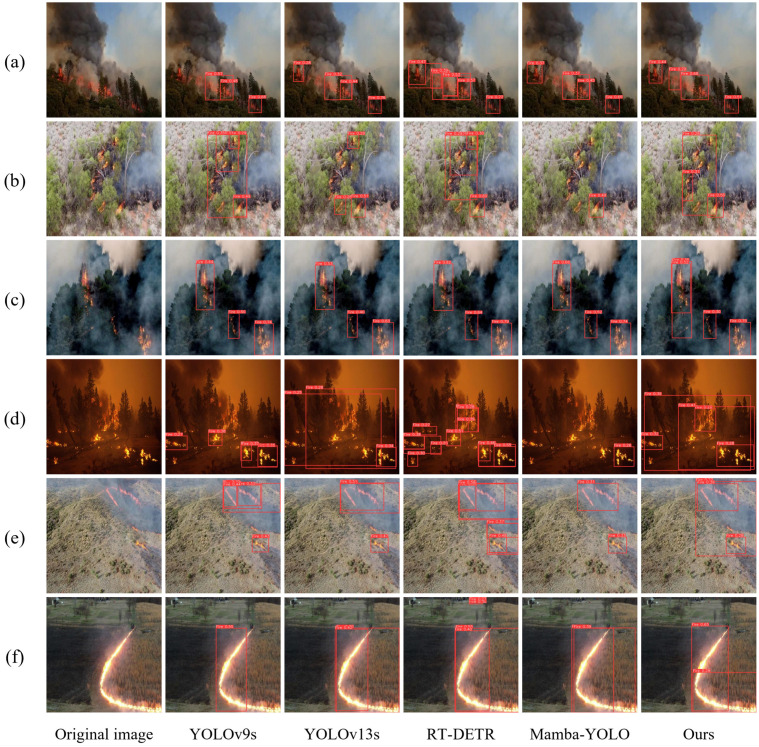
Visual comparison between the proposed method and other models. (**a**–**c**) Multi-target fires with smoke and vegetation occlusion. (**d**) Multi-target fires in low-light. (**e**) Mountainous forest fires. (**f**) Grassland fires with arc-shaped flames.

To intuitively evaluate the actual performance of the FF-Mamba-YOLO model in remote sensing forest fire detection, representative scenarios were selected for comparative inference analysis. The detection results of various models under typical scenarios are shown in Figure 11.

As shown in Figure 11, in Figure 11a,e, due to smoke occlusion, YOLO series models and Mamba-YOLO all exhibit varying degrees of detection omissions. In contrast, RT-DETR and FF-Mamba-YOLO models successfully detected all targets. Figure 11b,c show cases where flame targets are occluded by vegetation against forest backgrounds. Under such conditions, target detection is hindered. In Figure 11b, YOLOv13s and Mamba-YOLO only detected partial targets, and RT-DETR also had omissions. Notably, FF-Mamba-YOLO is the only model that successfully detected all targets in both Figure 10b,c, and in Figure 11c, it identified tiny flames that were almost invisible to the naked eye, which is crucial for real-time and accurate forest fire detection. Figure 11d depicts low-light scenarios where the background color is similar to flames and targets are densely distributed. The comparison shows that YOLOv13s, RT-DETR, and FF-Mamba-YOLO perform better, successfully detecting all flames. Conversely, YOLOv9s and Mamba-YOLO had incomplete detection with obvious omissions. In Figure 11f, all five models detected the long arc-shaped flames; however, the RT-DETR model misidentified the red object above as a flame target, and such errors may hinder the normal operation of UAV fire monitoring tasks.

In summary, FF-Mamba-YOLO can address key challenges such as occlusion, multi-target scenarios, scale variations, and complex background interference, achieving effective detection in various complex scenarios with higher accuracy and robustness, well meeting the requirements for UAV forest fire detection.

#### 4.6.2. Grad-CAM Visualization Analysis

**Figure 12:**
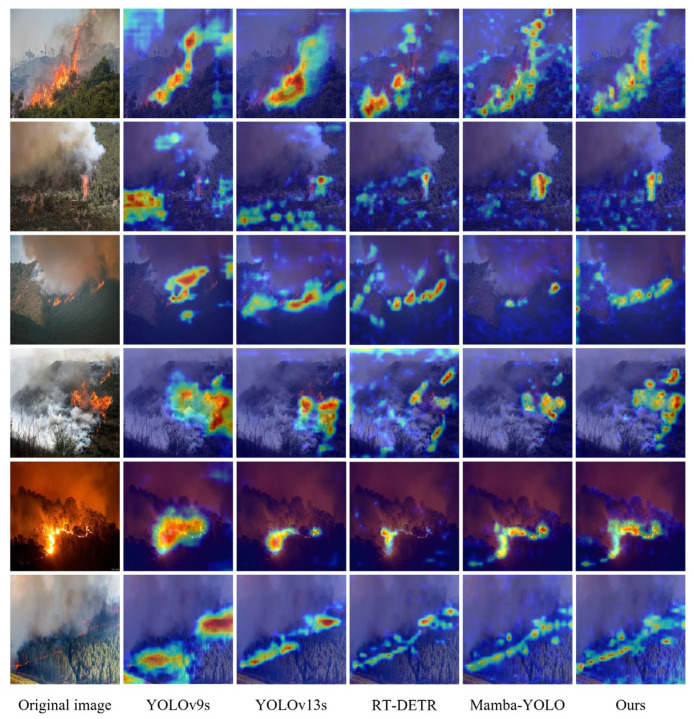
Grad-CAM generated heatmaps for fire targets utilizing YOLOv9s, YOLOv13s, RT-DETR, Mamba-YOLO, and FF-Mamba-YOLO.

To intuitively evaluate the effectiveness of the improved algorithm, this study employs gradient-weighted class activation mapping (Grad-CAM) to generate heatmaps and analyze the model’s attention regions, determining whether the model has learned the correct features. Figure 12 displays the heatmaps of different models, where red regions indicate higher model attention.

The heatmaps reveal that FF-Mamba-YOLO directs more precise attention toward target regions compared with other models. Specifically, in complex background environments, FF-Mamba-YOLO can focus on target regions while effectively suppressing the influence of background noise or other interference, successfully overcoming false detection issues. Simultaneously, when faced with vegetation and smoke occlusions, FF-Mamba-YOLO can accurately capture and enhance features of key regions, thereby accurately identifying fires.

Furthermore, FF-Mamba-YOLO demonstrates excellent multiscale adaptation capabilities when processing targets of different sizes. For larger-scale targets, our FF-Mamba-YOLO model can precisely focus on the entire fire-affected area. For tiny targets, despite occupying minimal regions, FF-Mamba-YOLO can still accurately perceive these critical areas, effectively overcoming missed detection issues. Notably, for targets with elongated shapes and unclear boundaries, FF-Mamba-YOLO can precisely capture target contours, significantly improving regression accuracy.

The visualization results further validate the model’s effectiveness in feature extraction and interference suppression, as well as its excellent adaptability and robustness in identifying targets of different scales. This intuitively demonstrates the superior performance of FF-Mamba-YOLO in remote sensing image fire detection tasks.

## 5. Discussion

In recent years, deep learning has rapidly advanced, demonstrating powerful capabilities in forest fire detection from remote sensing imagery. However, while current methods achieve high accuracy and robustness, they may introduce substantial redundant information when processing multiscale features and complex spatial relationships, leading to excessive computational burden that compromises real-time performance.

Recent research work [50] has also attempted to apply Mamba-based approaches to address general object detection problems. The findings of [51] are consistent with the results of this study, further demonstrating the feasibility of Mamba-fused solutions for object detection tasks. This paper proposes an effective framework based on Mamba and YOLO for forest fire detection. It represents a significant exploration of integrating the emerging Mamba into remote sensing forest fire monitoring, providing new insights for the development of deep learning-based remote sensing object detection technologies. Its optimal detection performance and model complexity demonstrate its suitability for practical applications.

In remote sensing image fire detection tasks, the proposed FF-Mamba-YOLO framework demonstrates significant improvements in Precision, mAP@50, and mAP@50:95 metrics, but limitations remain in Recall, potentially missing some fire instances in certain scenarios. Compared with lightweight models such as YOLOv8n, the computational cost of the model remains relatively high, which may limit its deployment in large-scale forest fire monitoring. While the UFFD exhibits diversity, it remains limited compared with large-scale public benchmarks, with potentially insufficient representation of rare or extreme weather conditions (e.g., fog, rain, heavy smoke with low visibility). Moreover, the UFFD dataset contains only RGB images.

Moving forward, to further push the boundaries of lightweight object detection and improve the model’s recall capability, we plan to investigate other hybrid models based on state space models and optimize structural design to balance accuracy and efficiency. Furthermore, we plan to expand the UFFD to include rare and extreme scenarios to enrich samples under different conditions, ensuring broader generalization capabilities. In addition to dataset expansion, we will explore multimodal fusion approaches in the future, such as combining RGB images with thermal infrared images, to enhance the accuracy and robustness of forest fire detection by integrating visual and thermal features.

## 6. Conclusions

This study introduced FF-Mamba-YOLO, a novel framework for UAV remote sensing forest fire detection, designed to overcome the challenges posed by multiscale targets and complex environmental interference in UAV remote sensing images. Furthermore, to address the limited availability of existing remote sensing data, we created the UFFD. The Mamba-based modules in the proposed model enable effective long-range dependency modeling while reducing computational complexity. The integration of CFEBlock and MGBlock into the Mamba-based modules effectively enhances the model’s capability for complex feature processing, enabling the model to effectively capture key features in multiscale environments. The enhanced neck structure combined with DySample further improves feature fusion quality, thereby demonstrating stronger robustness and reliability in real forest fire scenarios. Ablation studies and comparative analysis further validate the superior performance of FF-Mamba-YOLO in remote sensing image fire detection tasks, particularly in scenarios with dense distributions, complex backgrounds, and extreme scale variations. However, it also has limitations: the improvement in Recall is limited, and the relatively high computational overhead may affect its large-scale deployment. In future work, we will integrate more advanced lightweight architectures and image enhancement preprocessing modules to further improve the model’s detection accuracy and generalization capabilities. Simultaneously, we will further expand the dataset and employ generative adversarial networks to enhance dataset diversity. Additionally, we will investigate the fusion of multimodal sensor data to enhance the model’s robustness under challenging operational conditions. This work holds significant importance and application prospects for UAV-based forest fire detection tasks.

## Figures and Tables

**Table 1:** Experiment setup and training parameters.

Name	Value	Name	Value
Optimizer	SGD	Training Epochs	150
Initial Learning Rate	0.01	Batch Size	4
Weight Decay	0.0005	Workers	8
Momentum	0.937	Image Size	1024 × 1024

**Table 2:** Comparative results of different methods on the UFFD.

Model	Precision (%)	Recall (%)	mAP@50 (%)	mAP@50:95 (%)	Parameters (M)	GFLOPs
Faster R-CNN	54.3	66.4	62.0	29.7	28.3	157.0
YOLOv3-Tiny	55.9	61.0	59.9	28.6	12.1	18.9
YOLOv5s	60.9	58.5	63.1	32.6	9.1	23.8
YOLOv6s	61.3	62.0	64.4	34.3	16.3	44.0
YOLOv8n	57.1	61.2	62.7	32.9	3.0	8.1
YOLOv8s	59.8	58.8	63.1	33.4	11.1	28.4
YOLOv9s	62.2	61.9	65.1	35.7	7.2	26.7
YOLOv10s	60.2	59.5	62.8	33.5	8.0	24.4
YOLOv11s	58.6	63.1	64.8	34.4	9.4	21.3
YOLOv12s	58.0	62.6	64.3	34.6	9.1	19.3
YOLOv13s	59.6	61.9	65.3	34.8	9.0	20.7
RT-DETR	60.0	57.3	59.6	30.3	41.9	125.6
Mamba-YOLO	61.0	62.5	65.0	35.1	6.0	13.6
Ours	64.8	62.1	67.4	36.3	9.4	20.1

**Table 3:** Comparative experiments of different upsamplers.

Method	Precision (%)	Recall (%)	mAP@50 (%)	mAP@50:95 (%)	Parameters (M)	GFLOPs
Nearest	59.1	64.6	66.5	35.6	9.4	20.1
Bilinear	62.4	62.6	66.2	35.2	9.4	20.1
CARAFE	62.7	64.2	66.3	35.8	9.8	20.7
DySample	64.8	62.1	67.4	36.3	9.4	20.1

**Table 4:** Individual module ablation experiments.

CFE Block	MG Block	E-PAFPN	DySamlpe	Precision (%)	Recall (%)	mAP@50 (%)	mAP@50:95 (%)	Parameters (M)	GFLOPs
				60.7	61.1	64.2	33.7	3.7	8.1
✔				59.9	64.8	65.9	35.6	6.5	14.6
	✔			60.8	62.5	65.4	35.1	6.0	13.2
		✔		60.1	61.7	65.1	34.8	3.9	8.3
			✔	57.2	65.7	64.6	34.6	3.7	8.1

**Table 5:** Multi-module ablation experiments.

CFE Block	MG Block	E-PAFPN	DySamlpe	Precision (%)	Recall (%)	mAP@50 (%)	mAP@50:95 (%)	Parameters (M)	GFLOPs
				60.7	61.1	64.2	33.7	3.7	8.1
		✔	✔	62.1	63.0	65.7	35.1	3.9	8.3
✔	✔			60.4	64.4	66.2	35.4	8.8	19.6
✔	✔	✔		59.1	64.6	66.5	35.6	9.4	20.1
✔	✔		✔	61.2	62.7	66.2	35.8	8.8	19.6
✔	✔	✔	✔	64.8	62.1	67.4	36.3	9.4	20.1

**Table 6:** Comparison of different models on the FlameVision dataset.

Model	Precision (%)	Recall (%)	mAP@50 (%)	mAP@50:95 (%)	Parameters (M)	GFLOPs
YOLOv9s	86.5	88.9	94.2	72.3	7.2	26.7
YOLOv13s	88.4	88.2	94.6	72.2	9.0	20.7
Mamba-YOLO	88.9	87.6	94.8	71.7	6.0	13.6
Ours	90.8	88.4	95.5	72.4	9.4	20.1

## Data Availability

The data presented in this study are available on request from the corresponding author.
